# Decision-based interactive model to determine re-opening conditions of a large university campus in Belgium during the first COVID-19 wave

**DOI:** 10.1186/s13690-022-00801-w

**Published:** 2022-03-04

**Authors:** Vincent Denoël, Olivier Bruyère, Gilles Louppe, Fabrice Bureau, Vincent D’orio, Sébastien Fontaine, Laurent Gillet, Michèle Guillaume, Éric Haubruge, Anne-Catherine Lange, Fabienne Michel, Romain Van Hulle, Maarten Arnst, Anne-Françoise Donneau, Claude Saegerman

**Affiliations:** 1 Structural & Stochastic Dynamics, Faculty of Applied Sciences, University of Liège, Belgium, Allée de la découverte 9, B-4000 Liège, Belgium; 2 WHO Collaborating Centre for Public Health Aspects of Musculo-Skeletal Health and Ageing, Liège, Belgium; 3 Research Unit in Public Health, Epidemiology and Health, Economics, University of Liège, Quartier Hôpital, Av. Hippocrate 13, CHU B23, 4000 Liège, Belgium; 4 Montefiore Institute, Faculty of Applied Sciences, University of Liège, Allée de la Découverte 10, B-4000 Liège, Belgium; 5 Laboratory of Cellular and Molecular Immunology, GIGA Institute, ULiège, 4000 Liège, Belgium; 6 Faculty of Veterinary Medicine, ULiège, 4000 Liège, Belgium; 7 Walloon Excellence in Life Sciences and Biotechnology (WELBIO), Wallonia, Belgium; 8 Research Unit in Emergency Medicine, Faculty of Medicine, University of Liège and University Hospital of Liège, Liège, Belgium; 9 Institute for Research in Social Sciences (IRSS), Faculty of Social Sciences, University of Liège, Place des Orateurs, 3, B-4000 Liège, Belgium; 10 Laboratory of Immunology-Vaccinology, FARAH, ULiège, 4000 Liège, Belgium; 11 Research Unit in Biostatistics and Research Methods, University of Liège, Quartier Hôpital, Av. Hippocrate 13, CHU B23, 4000 Liège, Belgium; 12 Terra Research Center, Gembloux AgroBioTech, University of Liege, Passage des Deportes, 2, B-5030 Gembloux, Belgium; 13 Récolte et Analyse de Données et d’Information d’Utilité Stratégique (RADIUS), University of Liège, Place du 20-Août, 7, 4000 Liège, Belgium; 14 Computational and stochastic modelling, Faculty of Applied Sciences, University of Liège, Allée de la découverte 9, B-4000 Liège, Belgium; 15 Fundamental and Applied Research for Animal and Health (FARAH) Center, Liège University, Liège, Belgium

**Keywords:** Screening, COVID, Pandemic, Model, University, Student

## Abstract

**Background:**

The role played by large-scale repetitive SARS-CoV-2 screening programs within university populations interacting continuously with an urban environment, is unknown. Our objective was to develop a model capable of predicting the dispersion of viral contamination among university populations dividing their time between social and academic environments.

**Methods:**

Data was collected through real, large-scale testing developed at the University of Liège, Belgium, during the period Sept. 28th-Oct. 29th 2020. The screening, offered to students and staff (*n* = 30,000), began 2 weeks after the re-opening of the campus but had to be halted after 5 weeks due to an imposed general lockdown. The data was then used to feed a two-population model (University + surrounding environment) implementing a generalized susceptible-exposed-infected-removed compartmental modeling framework.

**Results:**

The considered two-population model was sufficiently versatile to capture the known dynamics of the pandemic. The reproduction number was estimated to be significantly larger on campus than in the urban population, with a net difference of 0.5 in the most severe conditions. The low adhesion rate for screening (22.6% on average) and the large reproduction number meant the pandemic could not be contained. However, the weekly screening could have prevented 1393 cases (i.e. 4.6% of the university population; 95% CI: 4.4–4.8%) compared to a modeled situation without testing.

**Conclusion:**

In a real life setting in a University campus, periodic screening could contribute to limiting the SARS-CoV-2 pandemic cycle but is highly dependent on its environment.

## Background

The coronavirus disease 2019 (COVID-19) pandemic has created public health and social concerns globally, touching all parts of society. In September 2020, re-opening universities was a particular challenge due to the high likelihood of contagion within this particular population. In fact, during face-to-face teaching, infected students could have transmitted the virus to other students and staff members, who could have, in turn, infected students in other classes [1]. Indeed, transmission could occur in classroom settings particularly where there was an inappropriate use of face masks, insufficient physical distancing, poor ventilation or inadequate hand hygiene. In addition, there was the presence of collective living environments and the difficulty of limiting socialization and group gatherings outside classes and even outside the university campus. In fact, it could be hypothesized that student gatherings and collective living spaces, both on and off campus, contributed to the rapid spread of SARS-coV-2 on campus. A study conducted in August in the United States [2] reported a rapid increase in COVID-19 cases just 2 weeks after re-opening the university to students.

Research carried out in American residential college campuses suggested that symptom-based screening alone was not sufficient to contain an outbreak and to allow students to work safely [3]. Indeed, knowing that a significant proportion of infections would be asymptomatic, it was crucial to prevent large outbreaks on and off campus and to detect and isolate infections as they occurred. In an attempt to reduce transmission, Walke et al. suggested a number of possible options [4]: (a) testing all students before arrival on campus; (pre-arrival testing paired with a follow-up test); (b) repeated testing of the entire campus population; (c) testing a random sample of the campus population; (d) making tests available to students on campus on demand but not mandatory. The latter option was applied to the University of Liège (ULiège) in Belgium, by means of a massive undertaking for saliva testing.

Considering the fact that allowing students to work in a traditional way was not acceptable, reopening risk mitigation plans were developed in most of the Universities. These plans generally included prevention practices, mitigation measures and testing strategies. At ULiège, in addition to classical mitigation measures, a mass screening program was available 2 weeks after the University re-opened. Interestingly, ULiège is located in Belgium, a country where COVID-19 prevalence was significantly higher than neighboring countries at the moment of this study. Moreover, since ULiège students also have significant interaction with the city’s population, a specific analysis of the efficiency of the screening strategy was necessary.

Various models of the dynamics and spread of COVID-19 have been reported in the current literature. Most of these were initially based on the Susceptible-Exposed-Infected-Removed (SEIR) model or the Susceptible-Infected-Recovered (SIR) model, as exhaustively reviewed [5]. As of today, more advanced models incorporating the heterogeneity in the population are available, e.g. [6], in particular in Belgium [7, 8]. The authors noted that mathematical modeling has shown to be a reliable tool in the fight against this pandemic. However, it was suggested that due to the substantial uncertainty surrounding the multiple inputs to the model, it was prudent to explore a range of plausible scenarios that would present a wide range of results [9]. Even if it precluded accurate projections of future results, university authorities can use this data to assist risk management by means of uncertain propagation and worst-case scenarios.

By feeding the results of the mass screening organized at ULiège into a mathematical compartmental model, adjusting the model parameters to the data and simulating alternative control strategies, it was possible to quantify the real impact of the periodic testing strategy on the control of COVID-19.

## Methods

Screening was set up at ULiège to monitor the development of the pandemic during the fall 2020 semester, targeting both students and staff. The population was estimated to be 30,000, representing the total number of individuals (80% students, 20%staff) offered testing. It is an average value of the weekly size of the population over the testing period. Saliva was tested, with a sensitivity of 65% (and 85% in some additional cases) and a specificity of 99% (﻿in a later stage of the epidemics, where reported cases were very low, saliva testing was continued and over several days in a row, groups of more than hundred individuals all tested negative, which indicates that the saliva testing did not detect false positives; the specificity was therefore estimated to be larger than 99%.). Results were usually available within 24 h of self-sampling. Screening was organized on a voluntary and weekly basis from September 28th (2 weeks after the start of courses) to October 29th. Testing was anonymous. Individual results were obtained by introducing the unique barcode on the testing kit onto a specific secure interface powered by the covid-19 Diagnostic Platform of ULiège. In this interface, each university member was asked to enter details such as their profile (student or staff) and local affiliation, in order to allow provisional analysis of the data. The results associated with each group were analyzed separately but also integrated and formatted in a global report generated daily by the institutional reporting group and communicated to the internal risk assessment group and risk management group.

**Fig. 1:**
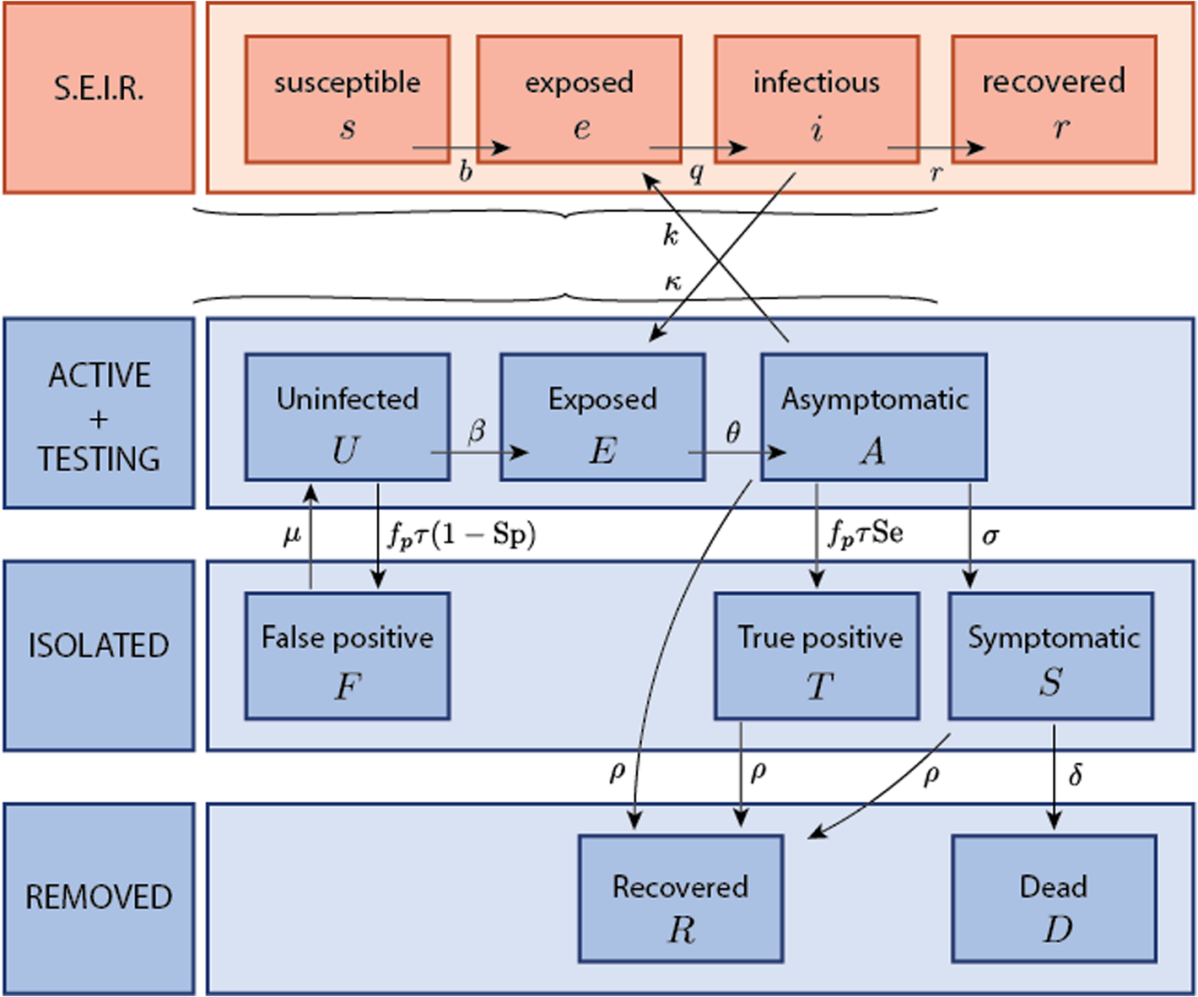
Graphic representation of the two-population model. A Susceptible-Exposed-Infected-Removed (SEIR) model was used for the external population (in red). An extended SEIR model was used for the university population (in blue). Arrows indicate transitions between compartments. In particular *κ*/*β* represents the relative importance of the university population mixing with the urban population

Analysis of the data was carried out from three perspectives:

1. Extensive descriptive statistics by means of the automatic reporting described above gave regular up-dates.
2. A two-population compartmental model, based on the model proposed by Paltiel [3], was conceived to fit the situation. It consisted of a standard SEIR model representing the external population (the city and province of Liège) together with an 8 state-model representing the different compartments within the university (tested). The model is depicted in Fig. 1; its full description and governing equations are available in Appendix 1. A number of biological characteristics of the pathogens of the pandemic were included (Table 1), as well as some parameters related to the screening such as participation levels and testing frequency. Other model parameters including the reproduction number and transmission rates were specific to the pandemic at a given time and location. These parameters of the model were therefore inferred from the collected data. Specifically, a multiple output nonlinear regression [10] was implemented to fit the model observations (number of cases in the population and, more importantly, cumulative number of positive saliva tests). Best-fit and 95%-confidence intervals (95%-CI) of the reproduction numbers characterizing the two populations were obtained at key dates during the screening period. It is underlined that the reproduction number used in this model should not be confused with the global indicator such a that reported on a daily basis by Sciensano (which encompasses many more aspects of the pandemics). In this model, it is just related to the transmission rate parameter β, so it is natural that it takes slightly different values that those reported in the media. ﻿In addition, the reproduction number depends on the mitigation measures implemented in a specific location and community, which also explains why the values we obtained for the University of Liège assume different values that in the general population. The exogenous contamination of the external population by the university population was quantified in different scenarios. This was done by selecting several values of the coupling index referring to the relative magnitude of the fluxes escaping the uninfected compartment (U) because of exogenous and endogenous contamination respectively, see details in Appendix 1.
  The most uncertain parameters of the model were those related to cross-contamination. Three scenarios were therefore considered: (A) population size ratio *n*_*E*_/*n*_*U*_ = 380 and no coupling (*κ*/*β* = 0), (B) population size ratio *n*_*E*_/*n*_*U*_ = 20 and coupling ratio *κ*/*β* = 0.25, (C) population size ratio *n*_*E*_/*n*_*U*_ = 20 and coupling ratio *κ*/*β* = 0.50. These scenarios were specifically chosen to represent either (A) the whole population in Belgium as an external population *n*_*E*_ = 11.4*e*6 individuals, or (B-C) the estimated population surrounding the university campuses *n*_*E*_ = 600*e*3 individuals, while varying the coupling ratio in a significantly large and representative range. Endogenous contaminations are population-specific (e.g. social distancing) parameters identified by means of the least-square fitting of the model; they were expressed by means of the effective reproduction numbers *r*_*T*_ and *R*_*T*_ in the two populations, see Table 1, and were assumed to vary in a stepwise manner between the key dates related to the federal management of the crisis: (i) on October 6th, physical contact was restricted; e.g. bars had to close at 11:00 pm; (ii) October 19th, a night-time curfew was introduced, (iii) November 2nd, national lock-down. These three key dates define the three time frames.
3. finally, the influence of the participation rate, the start of the screening and frequency of the testing were investigated. This was achieved by simulating alternative configurations that would have taken place under different control strategies.

**Table 1:** Main parameters of the model and their numerical values. In particular, transmission rates in urban and university populations are expressed by means of the reproduction numbers *r*_0_ and *R*_0_ in these two populations, respectively

Symbols		Type	Units	Values
Se	Test sensitivity	Fixed	[−]	0.65
Sp	Test specificity	Fixed	[−]	0.99
*μ*	Return Rate of false positive	Fixed	[day^−1^]	1/3
*θ*	Rate of advance to asymptomatic	Fixed	[day^−1^]	1/3
*ρ*	Rate of recovery	Fixed	[day^−1^]	1/14
*f*_*s*_	Rate of symptom development	Fixed	[%]	30%
*f*_*r*_	Fatality risk	Fixed	[−]	0.0005
*β*, *κ*, *b*, *k*	Transmission rates (exogenous and endogenous)	Variable	[day^−1^]	[0.03–0.6]
*r*_*T*_	Reproduction number in external population	Identified	[−]	[0.6–3.5]
*R*_*T*_	Reproduction number in university population	Identified	[−]	[0.6–3.5]
*n*_*E*_	Size of external population	Variable	[indiv.]	{11.4e6; 0.6e3}
*n*_*U*_	Size of university population	Fixed	[indiv.]	[30,000]
*τ*	Testing frequency	Variable	[day^−1^]	{0, 1/7, 2/7}
*f*_*p*_	Participation rate	Variable	[%]	[0–100%]

These steps rely on the development of the two-population model and its implementation. The source code programmed in Matlab is available from the authors’ institutional repository [11].

## Results

1. Descriptive statistics

With a total number of 41,021 screening tests performed over the 5-week period, the actual average participation rate was 22.6%. Unfortunately, the rapidly developing pandemic in Belgium at the time of the screening (October 2020) did not allow the testing to control the situation, e.g. by dropping the reproduction number below unity. The screening had to be halted after a period of 5 weeks, when the campus and the whole country entered a lock-down implemented by the government to react to the uncontrolled nature of the situation. During the last two testing weeks, 4.31% and respectively 7.12% of the tests were positive, which was the initial indicator of the severity of the situation.

2. The mathematical model: parameter fitting

The model parameters were adjusted after the initial findings: the fraction of infectious individuals in the external population (estimated from the new daily-declared cases, see Appendix 2) and the number of positive results from the test period organized for the university population.

**Fig. 2:**
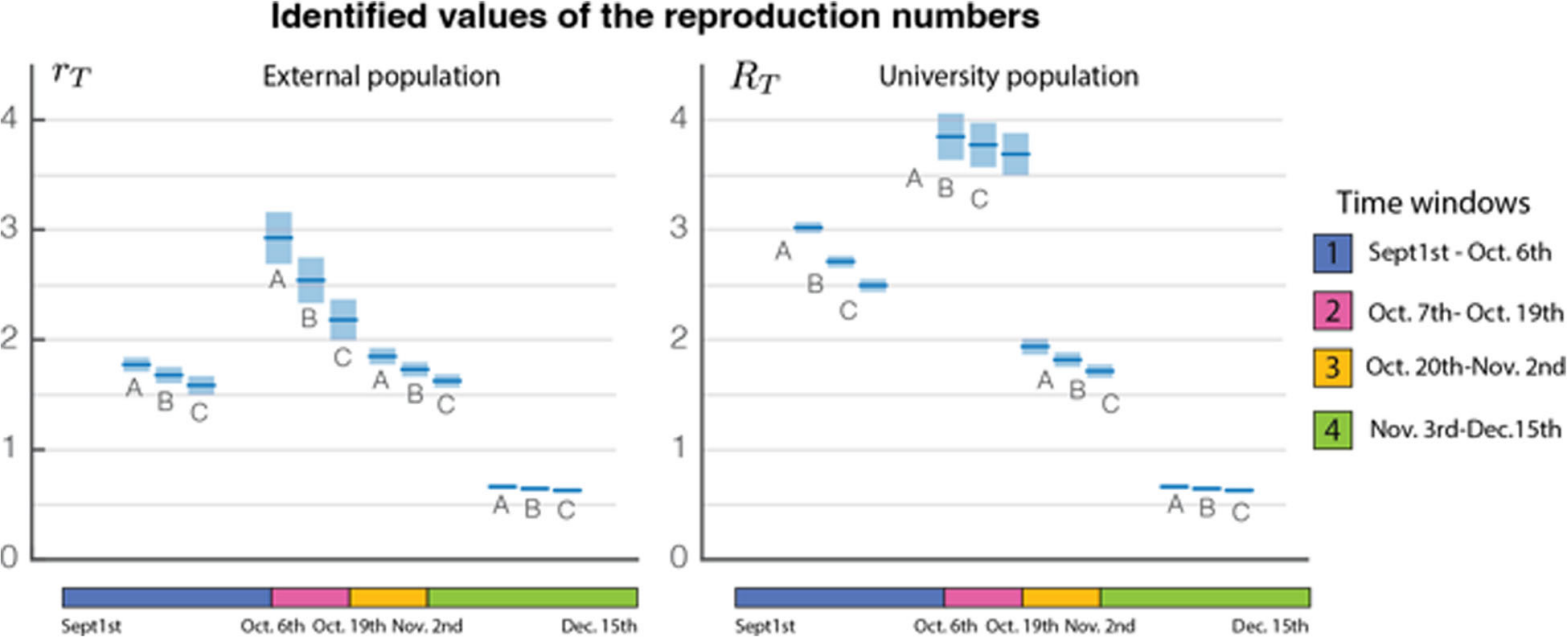
Illustration of the identified reproduction numbers *r*_0_ and *R*_0_, and their 95%-Cis in Belgium during the first COVID-19 wave. Symbols A, B and C refer to three scenarios. Numerical values, see Table 2

The identified values of the reproduction numbers are reported in Table 2 for each scenario and for each time frame, together with the 95%-CIs. They are also shown in Fig. 2. They correspond to the regression estimates and the symmetrical confidence interval obtained with the *nlinfit* function of Matlab (computed from the mean square error and Jacobian of the model). The nonlinear regression was based on the Levenberg-Marquardt algorithm. Error estimates on predictions were computed with the same toolbox (*nlpredci*). Although a slight compensation was observed with respect to the coupling and population size ratio, the identified reproduction numbers were only marginally dependent on the chosen scenario. There was a significant difference between reproduction numbers in the two populations: before the second key date (October 19th), it peaked at 3.5 throughout the campus compared to 2.7 in the external population (scenario B), see Table 2. Due to the early end of testing, the reproduction numbers in the university population along the last two time frames were assumed to mirror those of the external population.

**Table 2:** Values of the effective reproduction number identified in the 3 cross-contamination scenarios in a large university campus in Belgium during the first COVID-19 wave. The coupling index *κ*/*β* quantifies the importance of exogenous contamination in the university population; the population ratio corresponds to a coupling with the Belgian population (A) or with a regional population (B-C). The university population is *n*_*U*_ = 30,000 individuals

Scenario	Coupling index	Population ratio	Identified reproduction numbers in the different time windows			
			Sept. 1st-Oct. 6th	Oct. 7th-Oct. 19th	Oct. 20th-Nov. 2nd	Nov. 3rd-Dec.15th
A	0	380	1.77 [1.71; 1.83]	2.92 [2.70; 3.15]	1.85 [1.77; 1.92]	0.66 [0.64; 0.68]
			3.02 [2.97; 3.07]	3.85 [3.64; 4.06]	1.85 [1.77; 1.92]	0.66 [0.64; 0.68]
B	0.25	20	1.67 [1.60; 1.75]	2.54 [2.33; 2.75]	1.73 [1.66; 1.79]	0.64 [0.62; 0.66]
			2.71 [2.66; 2.76]	3.77 [3.57; 3.98]	1.73 [1.66; 1.79]	0.64 [0.62; 0.66]
C	0.5	20	1.58 [1.50; 1.67]	2.18 [1.99; 2.37]	1.62 [1.56; 1.68]	0.62 [0.60; 0.65]
			2.49 [2.44; 2.55]	3.69 [3.49; 3.88]	1.62 [1.56; 1.68]	0.62 [0.60; 0.65]

**Fig. 3:**
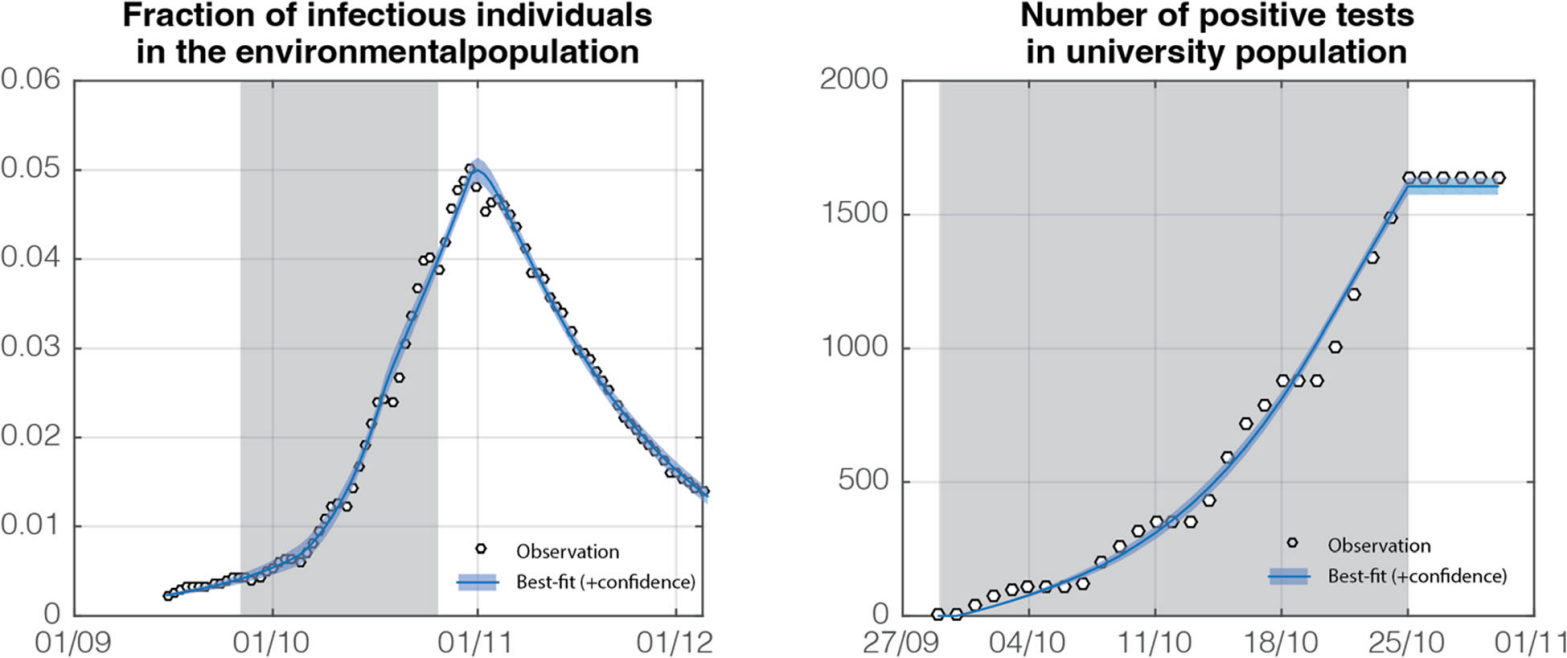
The best-fit and the 95%-CI of the model output (scenario B) around a large Belgian university during the first COVID-19 wave. The greyed zone corresponds to the time window during which the screening has been organized

The best-fit and 95%-CI of the model output are represented in Fig. 3. The agreement is remarkable in light of the simplicity of the model (only 6 parameters are adjusted) and, thanks to the homogenization of the population, the considered two-population compartmental model was found to be sufficiently versatile to capture the observed dynamic of the pandemic. The only significant discrepancy between findings and the model predictions was related to the stepwise nature of the cumulated number of tests (no analysis was performed at week-ends) while in reality it was smooth.

The continuously growing number of infectious individuals during screening seems to indicate no real stabilization effect. In fact, the large reproduction numbers meant it was impossible to contain the pandemic. With weekly testing, a preliminary design had shown that this screening was unable to control the pandemic with a reproduction number larger than *R*_*T*_ = 1.3, which was unfortunately the case at the start of the screening itself. The delay of 3 weeks between university re-opening and the start of screening was also detrimental.

3. Simulation of alternatives of scenario B

**Fig. 4:**
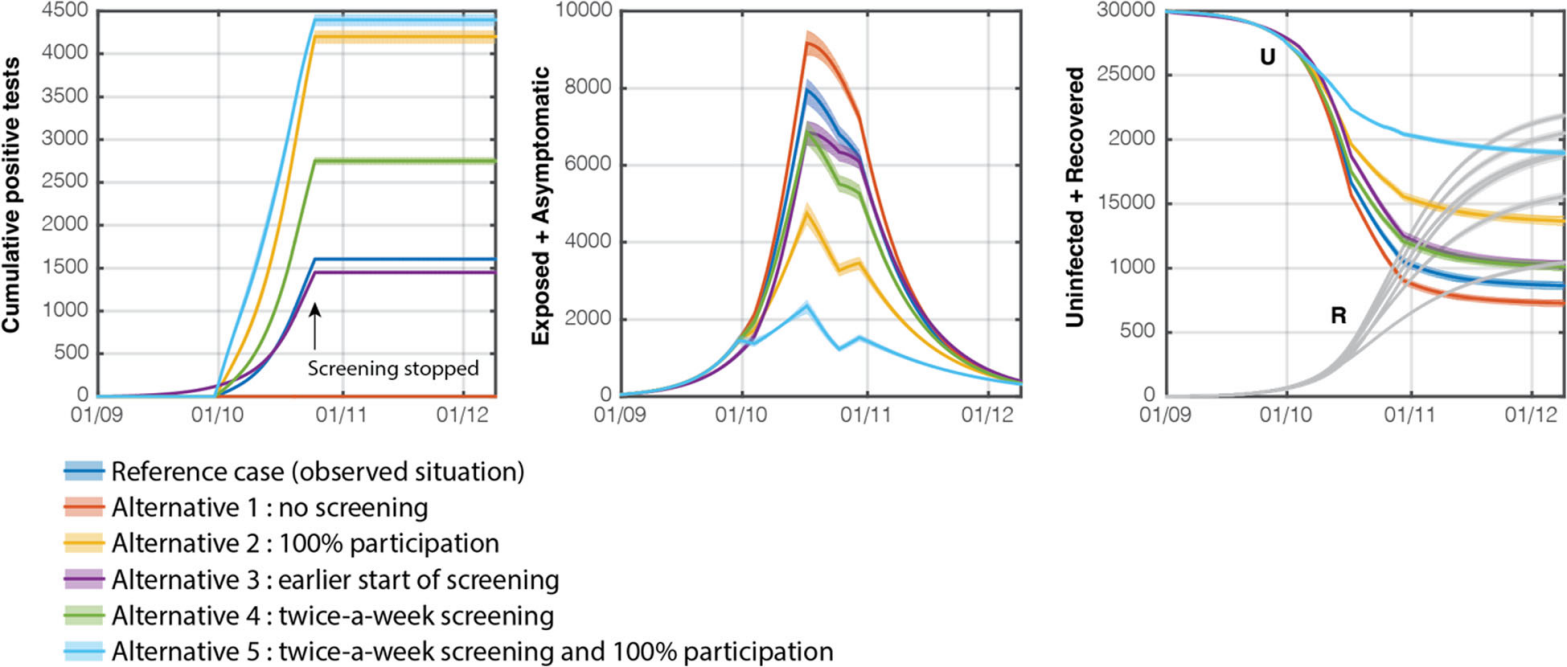
Evolution of the pandemic as alternatives to the observed situation in anuniversity campus in Belgium during the first COVID-19 wave. From left to right: (a) cumulative number of positive results over the screening period, (b) number of exposed and asymptomatic individuals in the university population, indicating the number of people who were isolated after screening (c) number of uninfected individuals in the university population and number of immune individuals (recovered). Units: number of individuals

The simulation of an alternative for scenario B revealed that weekly screening prevented 1393 cases (i.e. 4.6% of the university population; 95% CI: 4.4–4.8%) when compared to a modelled situation without testing. This estimation of prevented cases was obtained as the difference of contaminations 5 weeks after the end of screening (on December 3rd), based on numbers of uninfected individuals remaining in the university population, see Table 3. Several other alternatives with various modifications of the testing protocol have also been simulated and the savings have also been quantified for each of them, see Table 3 and Fig. 4. For instance, if screening had been started earlier on September 1st, 4409 cases would have been prevented, i.e. 14.7% of the population (95%CI 14.2–15.2%).

**Table 3:** Uninfected individuals in the Belgian university population on December 3rd (5 weeks after the end of screening) expressed as a number of individuals and fraction of population. The last column indicates the average net differences with the alternative 1 (no screening)

Alternative	Uninfected individuals in the university population on Dec. 3rd		Average Saving	
	Number of individuals	Fraction of population	Individuals	Fraction
Reference case (really tested situation)	8708 [8388; 9031]	29.0% [28.0, 30.1%]	1393	4.64%
Alternative 1: No screening	7315 [7007; 7613]	24.4% [23.4, 25.4%]	–	–
Alternative 2: 100% participation	13,732 [13,419; 14,060]	45.8% [44.7, 46.9%]	6417	21.4%
Alternative 3: Start screening on Sept. 1st	10,303 [9956; 10,649]	34.3% [33.2, 35.5%]	2988	10.0%
Alternative 4: Twice-a-week screening	10,189 [9863; 10,530]	34.0% [32.9, 35.1%]	2874	9.6%
Alternative 5: Twice-a-week screening and 100% participation	19,048 [18,837; 19,248]	63.5% [62.8, 64.2%]	11,733	39.1%

﻿Additional simulations carried out with a test sensitivity of 0.85 instead of 0.65 would have saved 1839 contaminations (instead of 1393) in the reference scenario. Likewise, in the other scenarios, the number of saved cases would increase: for instance, in Alternative 5 (combined measures scenario), 13,851 contaminations would have been prevented (instead 11,733). A meta-analysis [12] indicated that the sensitivity of saliva testing similar to the one used in this study was evaluated as Se = 0.95 [0.80–0.99]. The considered value Se = 0.65 therefore appears as a clearly safe lower bound. Sensitivity analyses, based on different parameters of the external population or on the sensitivity and the specificity of the screening test are presented in Appendix 3 and 4. While the specificity is seen to not affect the output of the model, the sensitivity has a slight influence on the results. However, the global trends are still there but they have more influence on the results than the parameters of the external population.

## Discussion

By comparing 5 alternative scenarios of the reference case, it was assessed whether, in our particular context, screening frequency and percentage of participation significantly affected the results obtained. In particular, screening twice a week would have prevented 2874 cases (i.e. 9.6% of the population) instead of 1393 (i.e. 4,6%). Interestingly, a full participation level by students and staff would have had an even more significant impact on the number of cases, by preventing 6417 cases (i.e. 21.4% of the population) with respect to a scenario without intervention. The combined action of total participation in a twice-a-week screening would have resulted in a more than additive performance, with a net saving of 11,733 individuals, i.e. 39.1% of the university population.

Lacking accurate information about the test sensitivity, additional simulations has been run with larger sensitivity The larger the test sensitivity, the more asymptomatic individuals are identified and isolated, which results in larger amounts of cases prevented. The announced savings can therefore be considered as lower bound estimates. As to the test specificity, results are insensitive to this parameter as it varies in the range [0.95–0.99].

**Fig. 5:**
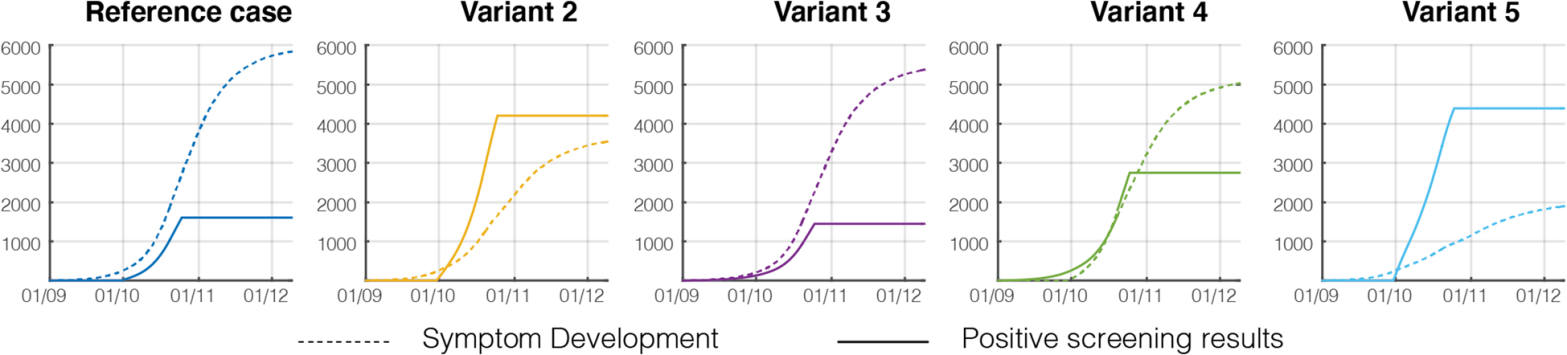
Comparison of the cumulated individuals extracted from the asymptomatic (transmission layer) compartment by means of either symptom development or positive screening results in an university campus in Belgium during the first COVID-19 wave. A 100% participation in screening (alternatives 2 and 5) shows a much better performance from this perspective

In our simplified model, there are two ways for asymptomatic individuals in the university population to commute to the isolation pool: either because of symptom development (transition to S), or thanks to detection after screening. Screening might be considered useful if it performs better than the symptom development in extracting individuals testing positive from the active transmission layer. In order to illustrate this, the cumulated fluxes out of the Asymptomatic compartment and to compartments Symptomatic and True Positive respectively are shown in Fig. 5, for each alternative. Dashed lines represent the influence of symptom development while the solid line represents screening. The screening in the alternatives 2 and 5, with 100% participation is seen to outperform the symptom development by a factor of 2 to 3, especially at early stages of screening. In the real tested situation, the fluxes through Symptomatic and True Positive were very similar during the screening period, which indicated an appreciable efficiency of screening.

In ULiège, according to the data collected, screening organized in the fall semester of 2020 was unable to curb the pandemic but could be considered as efficient as it was estimated that 1393 new cases were prevented (4.6% of the population) over the 5-week period. However, other key elements have shown to have an impact on reducing the incidence of COVID-19 positivity, including actual reproduction rates, frequency of screening and participation levels. The uniqueness of the University influenced a substantial part of the results and several factors must be taken into account to interpret them:

- First, in the fall of 2020, when the country was heavily impacted by COVID-19, the Province of Liège experienced one of the highest R_T_ in Belgium [13]. Screening at ULiège was originally designed at a time (spring-summer 2020) when reproduction numbers were not expected to reach such high levels, although another pandemic wave was forecast for September–October 2020. The magnitude of this wave was greater than anticipated and had a detrimental impact on the success of screening. In a study outlining how universities in Taiwan successfully re-opened, a key factor was that the country almost completely eliminated spread within the community first [14].
- Second, because of logistical constraints, screening was not possible before the official re-opening of the University but only 3 weeks after. However, universities that forced students to self-quarantine for 14 days before classes started have been shown to report lower than average levels of test positivity among students [15]. In the UK, the Independent SAGE Behavioural Advisory Group recommended that if students in the UK had to physically attend classes, there should be testing on or before arrival on campus. As in the UK we recommended screening as soon as possible, not only for students [16] but also for lecturers and other older campus staff [15] at higher risk of adverse effects from infection. It should be recognized that a single application of a screening test could miss cases in the early stages of infection, as well as cases that occur in the days between the result of the test and campus arrival.
- Furthermore, screening was not mandatory and consequently relied on the willingness of students and staff members to participate. The low level of participation in screening reduced the effectiveness of the results.
- Finally, because the screening test and results were anonymous, it was not possible to keep track of individuals who tested positive (notably to assess whether they respected quarantine requests when positive)..

At ULiège, as in other Belgian universities, it was recommended keeping face-to-face lectures to a minimum, in particular for large groups and, therefore, to give preference to online courses. Indeed, using transcription data from a mid-sized American university, infection rate was shown to decrease when large groups of 100 or more students were dropped from the network. The authors also suggested that mid-sized groups should also be withdrawn [1]. In our experience, and in agreement with others, hybrid teaching models can reduce but not eliminate the potential for the pandemic to spread. However, it is known that the potential for transmission of infection depends in part on factors that are under the control of students/staff and university administrators (e.g. obligation to wear a mask, physical distancing, auditorium capacity, ventilation). Enrolment in the same class did not capture all possible sources of contact between students [1]. It was then of primary importance to consider the environment in a global model.

Regardless, numerous mitigation measures were taken at ULiège to prevent the spread of COVID-19 on campus, which could have impacted the effectiveness of screening. A Task Force met regularly to discuss updates on the pandemic and modify existing or announce new measures as needed. For example, opening an auditorium to a maximum capacity of 50% to facilitate physical distancing, reducing and then closing dining rooms, providing a sanitizing gel or recommending the wearing of a mask and limiting direct social interactions. However, precise data on compliance with these important mitigation strategies was not available. In addition to the classic measures (i.e. social distancing, wearing a mask), it is imperative that students and staff adhere to the test schedule and (if the test is positive) isolation requirements.

Taiwan’s experience suggests that, under certain circumstances, safely re-opening colleges and universities this fall was feasible with a combination of strategies that included containment (access control with contact tracing and quarantine) and mitigation (hygiene, sanitation, ventilation, and social distancing) practices [14]. Therefore, a crucial step after screening is contact tracing with isolation of cases and quarantining of contacts. Unfortunately, due to the anonymous nature of screening, contact tracing was not possible in our institution. However, if isolation does not follow detection of infected students, testing is not effective. Interestingly, 2% of the students did not check their test results within 5 weeks of testing. Finally, while our university did not implement fully controlled contact tracing, isolation and quarantine procedures, because of the anonymous nature of the screening, a strong communication plan in favor of isolation and quarantine was provided by various sources. ﻿Anonymity also contributes to the bias in the results since, as only the fraction of participation in the screening is known, it is not possible to know whether the same group of persons participate in the screening or not, from week to week. It is likely that bias affects every single alternative studied in the paper in a similar manner. The announced number of saved contaminations (a difference of contaminations in two scenarios) is less sensitive to bias than the absolute number of contamination in each scenario. The saving of contaminations should correspond, at least approximatively, to some reality.

The model and data used in this study have both strengths and limitations. A strength of our study is that our models were guided by up-to-date disease transmission dynamics. However, of course, by way of limitation, the choices of the parameters included could have had a substantial impact on the dynamics of the infections and on the confidence in the results. Sensitivity analyzes were carried out to take this into account. Another advantage was that the model took both student population and university staff into account. However, a homogeneous transmission by age was assumed which may not reflect the accuracy of transmission. Since our screening program was anonymous, age could not be included in the model. We also used a two-population model, as an outbreak within the city population could also influence the pattern of disease transmission in the university community. The SEIR model for the external population is certainly too simple compared to the state-of-the-art (7a, 8a), but constitute a first amelioration of the exogenous contamination proposed in [5]. However, precise interaction between students, staff and the general population is complex, especially in our university with numerous campus locations. Finally, our model showed that the participation level is an important aspect in reducing the burden of transmission of COVID-19, but the exact proportion of participants was not known due to the hybrid method of teaching with some students or members of staff studying or working from home.

## Conclusion

In conclusion, it was observed that screening is a possible way of controlling the spread of the virus, but should be used for low to moderate values of the reproduction number. In more hostile situations, it can only limit damage. This paper has reported real large-scale testing and its simulated variations, which is innovative with respect to the literature dealing with mathematical models. The lessons learned from this testing are (i) do not hesitate to overdesign screening, i.e., making it more efficient than needed. This will compensate for unknown R_T_ and r_T,_as well as possible interactions with an environment, and contribute to the effectiveness of the screening, (ii) communicate with the tested population, so that the testing is taken up by all participants and does not jeopardize the efficiency of the screening. It highlights the need for robust and enhanced implementation of mitigation efforts and the need for additional mitigation measures specific to the setting. Finally, although it might not be able to fully control the pandemic, periodic screening can significantly contribute to reducing the number of infected people, and consequently casualties and fatalities.

## Data Availability

All analysis code and data are available upon request.

## Appendix 1

### Description of the mathematical model

The considered model is depicted in Fig. 1. The two populations are represented by different colors. The model for the urban population was based on a classical SEIR model^5^. The model for the controlled university population was an 8-state model^3^ broken down into three interconnected layers: the active and testing pool, the isolated pool and the removed pool. The model was borrowed from the literature^3^. It included several parameters collected in the nomenclature reported in Table 4 for clarity. While in the original model the isolation pool corresponded to an isolation dorm on the campus and the exogenous contamination in the active layer took place through weekly shocks (e.g. dorm parties with external people), a significant difference in the current model is the coupling with another population whose epidemic dynamics is also governed by a model.

**Table 4:** Nomenclature

Symbols	Signification
n	Index of time step
State variables	
*U*_*n*_, *E*_*n*_, *A*_*n*_	Number of individuals in the *active transmission* layer of the tested population (Uninfected, Exposed, Asymptomatic)
*s*_*n*_, *e*_*n*_, *i*_*n*_	Number of individuals in the *active transmission* layer of the large-scale model (susceptible, exposed, infectious)
*F*_*n*_, *T*_*n*_, *S*_*n*_	Number of individuals in the *isolation* layer of the tested population (False positive, True positive, Symptomatic)
*R*_*n*_, *D*_*n*_	Number of individuals in the *removed* layer of the tested population (Recovered, Dead)
*r*_*n*_	Number of individuals in the *removed* layer of the tested population
Parameters of the University model	
*n*_*U*_	Population size [individuals]
Se, Sp	Test sensitivity and specificity
β	Transmission rate (Asymptomatic → Uninfected) [day^−1^]
τ	Testing rate [day^−1^]
*f*_*p*_	Percentage of participation to testing [−]
μ	Rate of return to testing pool after isolation [day^− 1^]
θ	Incubation rate [day^−1^]
σ	Rate of symptom development [day^−1^]
ρ	Recovery rate [day^−1^]
δ	Death rate [day^−1^]
Parameters of the external population model	
*n*_*E*_	Population size [individuals]
b	Transmission rate (infectious → susceptible) [day^−1^]
q	Incubation rate [day^−1^]
r	Recovery + symptom development rate [day^−1^]
Coupling between the two populations	
κ, k	Cross-transmission rates [day^−1^]

The transmission rates *β*, *b* are transformed into reproduction numbers *R*_0_ and *r*_0_ to facilitate the interpretation of the numerical values by means of *β* = *R*_*T*_(*σ* + *ρ*) and *b* = *r*_*T*_*r*. They have the usual meaning of reproduction numbers in the uncoupled case.

The governing equations of the model can be conveniently provided in the form of the difference eqs. (A.1) and (A.2).

(A. 1)$$ \frac{U_{\left\{n+1\right\}}-{U}_{\left\{n\right\}}}{\varDelta t}=-\beta {\alpha}_{\left\{n\right\}}{U}_{\left\{n\right\}}-\left(1- Sp\right){f}_p\tau {U}_{\left\{n-1\right\}}+\mu {F}_{\left\{n\right\}}-\kappa {X}_{\left\{n\right\}} $$

$$ \frac{E_{\left\{n+1\right\}}-{E}_{\left\{n\right\}}}{\varDelta t}=-\theta {E}_{\left\{n\right\}}+\beta {\alpha}_{\left\{n\right\}}{U}_{\left\{n\right\}}+\kappa {X}_{\left\{n\right\}} $$

$$ \frac{A_{\left\{n+1\right\}}-{A}_{\left\{n\right\}}}{\varDelta t}=-\left(\sigma +\rho \right){A}_{\left\{n\right\}}-{f}_p\tau Se{A}_{\left\{n-1\right\}}+\theta {E}_{\left\{n\right\}} $$

$$ \frac{F_{\left\{n+1\right\}}-{F}_{\left\{n\right\}}}{\varDelta t}=-\mu {F}_{\left\{n\right\}}+\left(1- Sp\right){f}_p\tau {U}_{\left\{n-1\right\}} $$

$$ \frac{T_{\left\{n+1\right\}}-{T}_{\left\{n\right\}}}{\varDelta t}=-\left(\sigma +\rho \right){T}_{\left\{n\right\}}+{f}_p\tau Se{A}_{\left\{n-1\right\}} $$

$$ \frac{S_{\left\{n+1\right\}}-{S}_{\left\{n\right\}}}{\varDelta t}=-\left(\delta +\rho \right){S}_{\left\{n\right\}}+\sigma {T}_{\left\{n\right\}}+\sigma {A}_{\left\{n\right\}} $$

$$ \frac{R_{\left\{n+1\right\}}-{R}_{\left\{n\right\}}}{\varDelta t}=\rho {T}_{\left\{n\right\}}+\rho {A}_{\left\{n\right\}}+\rho {S}_{\left\{n\right\}} $$

$$ \frac{D_{\left\{n+1\right\}}-{D}_{\left\{n\right\}}}{\varDelta t}=\delta {S}_{\left\{n\right\}} $$

(A.2)$$ \frac{\left({s}_{\left\{n+1\right\}}-{s}_{\left\{n\right\}}\right)}{\varDelta t}=-b{a}_{\left\{n\right\}}{s}_{\left\{n\right\}}-{kx}_{\left\{n\right\}} $$

$$ \frac{\left({e}_{\left\{n+1\right\}}-{e}_{\left\{n\right\}}\right)}{\varDelta t}=-q{e}_{\left\{n\right\}}+b{a}_{\left\{n\right\}}{s}_{\left\{n\right\}}+{kx}_{\left\{n\right\}} $$

$$ \frac{\left({i}_{\left\{n+1\right\}}-{i}_{\left\{n\right\}}\right)}{\varDelta t}=-r{i}_{\left\{n\right\}}+q{e}_{\left\{n\right\}} $$

$$ \frac{\left({r}_{\left\{n+1\right\}}-{r}_{\left\{n\right\}}\right)}{\varDelta t}=r{i}_{\left\{n\right\}} $$

These translate the different fluxes from state to state in the model, see Fig. 1. In this set of equations, ∆t represents the time step used to integrate the first order delay differential equation. It was chosen equal to 1 day in the simulations reported in this document. The capital indexed symbols represent the number of individuals in the 8 states of the model. The lowercase index variables refer to the states of the external population, see Table 4.

Symbols ${\alpha}_{\left\{n\right\}}=\frac{A_{\left\{n\right\}}}{U_{\left\{n\right\}}+{E}_{\left\{n\right\}}+{A}_{\left\{n\right\}}+{R}_{\left\{n\right\}}}$ and ${a}_{\left\{n\right\}}=\frac{i_{\left\{n\right\}}}{s_{\left\{n\right\}}+{e}_{\left\{n\right\}}+{i}_{\left\{n\right\}}+{r}_{\left\{n\right\}}}$ respectively represent the fraction of asymptomatic individuals in the transmission layer of the controlled population and the fraction of infectious individuals in the external population. These ratios used together with the transmission rates *β*, *b, κ* and k model the transitions from Uninfected (susceptible) to Exposed (exposed) states. Notations are borrowed from the literature^3^ and, in particular, units in this system are individuals, i.e. all terms in the righthand side are rates of individuals (*β*, *τ*, *μ*, *κ*, *θ*, *σ*, *ρ*, *δ* in time^− 1^). The lowercase roman parameters *b, k, q, r* refer to rates having the same meaning as *β*, *κ*, { *θ*, *σ* } and *ρ* in the external population.

In the controlled population, the two states of the removed layer, namely the Recovered and Dead states are monovariant; the corresponding righthand sides are always positive. This is the same for the recovered state in the external population. They are the three absorbing states of the model.

The sum of all terms in the righthand sides is equal to zero, which guarantees that the total population is conserved and indicates that demography is neglected^14^. This model is aimed at focusing on a short prediction window, typically less than 1 year.

Three major differences between these equations and those presented in the literature^3^ concern

- the modeling of a contamination *κX*_{*n*}_ coming from another modeled population (instead of a lumped exogneous contamination, discussed next),
- the addition of parameter *f*_*p*_ (percentage of participation) which models the partial participation in the testing, in case of voluntary participation. A mandatory testing corresponds to the particular case *f*_*p*_ = 1;
- the fraction of asymptomatic individuals contributing to new contaminations is different from $\frac{A_{\left\{n\right\}}}{U_{\left\{n\right\}}+{E}_{\left\{n\right\}}+{A}_{\left\{n\right\}}}$ used in the literature^3^ which makes a substantial difference in simulations where the maximum number of asymptomatic individuals is large; in our model, it is deemed that recovered individuals *R*_{*n*}_ are reintroduced in the transmission layer. They are assumed to be immune, reducing therefore the rate of contamination.

The fluxes *κX*_{*n*}_ and *kx*_{*n*}_ corresponding to the transitions to the exposed states in each population and as a result of the mixing of the two populations are expressed by means of the coupling transmission rates *κ* and *k*. They take the form.

$$ \kappa {X}_{\left\{n\right\}}=\kappa \frac{i_{\left\{n\right\}}{U}_{\left\{n\right\}}}{s_{\left\{n\right\}}+{e}_{\left\{n\right\}}+{i}_{\left\{n\right\}}+{r}_{\left\{n\right\}}}=\kappa {a}_{\left\{n\right\}}{U}_{\left\{n\right\}} $$ and $$ k{x}_{\left\{n\right\}}=k\frac{A_{\left\{n\right\}}{s}_{\left\{n\right\}}}{U_{\left\{n\right\}}+{E}_{\left\{n\right\}}+{A}_{\left\{n\right\}}+{R}_{\left\{n\right\}}}=k{\alpha}_{\left\{n\right\}}{s}_{\left\{n\right\}} $$.

The model of the American campus^3^ is obtained as a particular case of this model where *κ* = *k* = 0.

## Appendix 2

### Estimation of the number of infectious individuals in the large population based on the number of confirmed cases

Authorities reported new daily cases on a regular basis. In the scope of the SEIR. model used for the large population, the cumulative of these reported cases corresponds to the fraction *f*_*s*._

(symptomatic people only) of infected and recovered individuals, i.e. *o*_{*n*}_ = *f*_*s*_(*i*_{*n*}_ + *r*_{*n*}_), where *o*_{*n*}_ corresponds to the cumulative of the number of observed cases. In the current model, a constant fraction *f*_*s*_ = 30% was assumed to model symptoms development; this simplifying assumption is similarly to developments in [3]. Substituting ${r}_{\left\{n\right\}}=\frac{o_{\left\{n\right\}}}{f_s}-{i}_{\left\{n\right\}}$ in the last eq. (A.2) yields

(A.3)$$ {i}_{\left\{n+1\right\}}-{i}_{\left\{n\right\}}=\varDelta t\left(-r{i}_{\left\{n\right\}}+\frac{o_{\left\{n+1\right\}}-{o}_{\left\{n\right\}}}{\varDelta t{f}_{\left\{s\right\}}}\right) $$

where *r* = *ρ* + *σ* = 0.102 day^− 1^ in this model. The difference *o*_{*n* + 1}_ − *o*_{*n*}_ corresponds to the daily new declared cases. This equation shows that the number of infectious individuals is obtained by convolving this difference with an exponential response function of characteristic time 1/*s*. The fraction of infectious individuals is then obtained by dividing by the total population, chosen equal to 11.4 ∙ 10^6^ since it represents to total population of Belgium during this pandemic.

## Appendix 3

Sensitivity analysis: number of saved contaminations, as a function of the parameters of the external population. SA-1: size of external population is 10 times (instead of 20 times) the size of university population. SA-2: size of external population is 100 times (instead of 20 times) the size of university population. SA-3: coupling coefficient is equal to 0.2 (instead of 0.25). SA-4: coupling coefficient is equal to 0.4 (instead of 0.25)

## Appendix 4

Sensitivity analysis: number of saved contaminations, as a function the test specificity (Sp) and sensitivity (Se). Original data: Se = 0.65, Sp = 0.99. SA-5: Se = 0.65, Sp = 0.90. SA-6: Se = 0.75, Sp = 0.99. SA-7: Se = 0.85, Sp = 0.99. SA-8: Se = 0.95, Sp = 0.99

## Abbreviations

COVID-19: Coronavirus disease 2019
ULiège: University of Liège
SEIR: Susceptible-Exposed-Infected-Removed
SIR: Susceptible-Infected-Recovered
95%-CI: 95%-confidence intervals

## References

[1] Weeden KA, Cornwell B (2020). The small-world network of college classes: implications for epidemic spread on a university campus. Soc Sci.

[2] Wilson E, Donovan CV, Campbell M, Chai T, Pittman K, Sena AC (2020). Multiple COVID-19 clusters on a university campus - North Carolina, august 2020. MMWR Morb Mortal Wkly Rep.

[3] Paltiel AD, Zheng A, Walensky RP (2020). Assessment of SARS-CoV-2 screening strategies to permit the safe reopening of college campuses in the United States. JAMA Netw Open.

[4] Walke HT, Honein MA, Redfield RR (2020). Preventing and responding to COVID-19 on college campuses. JAMA..

[5] Mohamadou Y, Halidou A, Kapen PT (2020). A review of mathematical modeling, artificial intelligence and datasets used in the study, prediction and management of COVID-19. Appl Intell.

[6] Davies NG, Klepac P, Liu Y, Prem K, Jit M, Eggo RM (2020). Age-dependent effects in the transmission and control of COVID-19 epidemics. Nat Med.

[7] Willem L, Abrams S, Libin PJ, Coletti P, Kuylen E, Petrof O, Møgelmose S, Wambua J, Herzog SA, Faes C, Beutels P, Hens N (2021). The impact of contact tracing and household bubbles on deconfinement strategies for COVID-19. Nat Commun.

[8] Abrams S, Wambua J, Santermans E, Willem L, Kuylen E, Coletti P, Libin C, Faes O, Petrof SA, Herzog PB, Hens N (2021). Modelling the early phase of the Belgian COVID-19 epidemic using a stochastic compartmental model and studying its implied future trajectories. Epidemics.

[9] Chang JT, Crawford FW, Kaplan EH. Repeat SARS-CoV-2 testing models for residential college populations. Health Care Manag Sci. 2020; 10.1007/s10729-020-09526-0 PMC7669307 33200374

[10] Chen. Multiple curve fitting with common parameters using NLINFIT. (https://www.mathworks.com/matlabcentral/fileexchange/40613-multiple-curve-fitting-with-common-parameters-using-nlinfit), MATLAB Central File Exchange Retrieved February 25, 2021 2021.

[11] ORBi, Open Repository and Bibliography, www.orbi.uliege.be, University of Liège.

[12] Saegerman C, Donneau AF, Speybroeck N, Diep AN, Williams A, Stamatakis L, et al. Repetitive saliva-based mass screening as a tool for controlling SARS-CoV-2 transmission in nursing homes. Transbound Emerg Dis. 2021; 10.1111/tbed.14280. Epub ahead of print. PMID: 34357691; PMCID: PMC8446975 10.1111/tbed.14280 PMC8446975 34357691

[13] Sciensano.be. Healthy all life long (Internet) [Available from: https://www.sciensano.be/en.]

[14] Cheng SY, Wang CJ, Shen AC, Chang SC (2020). How to safely reopen colleges and universities during COVID-19: experiences from Taiwan. Ann Intern Med.

[15] Yamey G, Walensky RP (2020). Covid-19: re-opening universities is high risk. BMJ..

[16] Independent SAGE Statement on universities in the context of SARS-CoV-2 [cited 2020 Sept 3]. Available from: https://www.independentsage.org/university_final_sept/.

